# Fabrication of Cellulose ZnO Hybrid Nanocomposite and Its Strain Sensing Behavior

**DOI:** 10.3390/ma7107000

**Published:** 2014-10-16

**Authors:** Hyun-U Ko, Seongcheol Mun, Seung-Ki Min, Gi-Woo Kim, Jaehwan Kim

**Affiliations:** 1 Department of Mechanical Engineering, Inha University, Incheon 402-751, Korea; E-Mails: lostmago@naver.com (H.-UK.); bobtf@hanmail.net (S.M.); msg8398@nate.com (S.-K.M.); 2 School of Automotive Engineering, Kyungpook National University, 386 Gajangdong, Sangju, Kyungsangpook-Do 742-711, Korea; E-Mail: gwkim2@knu.ac.kr

**Keywords:** cellulose, zinc oxide, piezoelectric, nanocomposite, strain sensor

## Abstract

This paper reports a hybrid nanocomposite of well-aligned zinc oxide (ZnO) nanorods on cellulose and its strain sensing behavior. ZnO nanorods are chemically grown on a cellulose film by using a hydrothermal process, termed as cellulose ZnO hybrid nanocomposite (CEZOHN). CEZOHN is made by seeding and growing of ZnO on the cellulose and its structural properties are investigated. The well-aligned ZnO nanorods in conjunction with the cellulose film shows enhancement of its electromechanical property. Strain sensing behaviors of the nanocomposite are tested in bending and longitudinal stretching modes and the CEZOHN strain sensors exhibit linear responses.

## 1. Introduction

Cellulose is a cheap and renewable biopolymer in the world, traditionally used for paper, pulp and textiles. In recent years, cellulose has been rediscovered as a smart material that can be used for sensors, actuators and electronics [1]. The electromechanical behavior of so-called electro-active paper (EAPap) has been reported and electromechanical devices, such as physical sensor, speaker, bending actuator have been demonstrated [2,3,4]. Besides, electrochemical and electrical devices based on cellulose hybrid composites have also been reported, such as gas sensor, pH sensor, biosensor, and field effect transistor [5,6,7,8]. 

Zinc oxide (ZnO) is a well known nanomaterial. Because of its wide band gap (3.47 eV) and high hole-electron biding energy (60 meV), great attention is paid to it for semiconductors, photo-catalytic devices, gas sensors, photovoltaics and piezoelectric energy harvesters [9,10,11]. Furthermore, since ZnO is biocompatible, it is useful for biomedical applications [12]. Micro energy harvesting devices is also a great issue of this material. Nanoscale mechanical energy can be converted into electrical energy by means of piezoelectric ZnO nanowire arrays [13]. On the other hands, composite materials of ZnO and organic material are very promising for flexible devices [10,11]. To fabricate the organic-ZnO composite material, suitable organic substrate is important. Because cellulose is a renewable material, it can be a great candidate for the organic substrate of ZnO hybrid composite. Some research efforts have been given for growing ZnO nanostructure on cellulose. A ZnO nanowire based solar cell was made on a flexible paper by sputtering ZnO [14]. ZnO strain sensor was made on cellulose substrate by hydrothermal method [15]. However, they only used cellulose paper as a flexible substrate. Since paper has randomly oriented cellulose fibers, the paper surface is so rough that its sensing performance could be low. 

In this paper, we report a fabrication of well-aligned ZnO nanorods on cellulose and its strain sensing behavior. To maintain good alignment of ZnO nanorods, a regenerated cellulose film was used, on which smooth surface is possessed, and a hydrothermal process was adopted with seeding and growing steps of ZnO. The fabricated CEZOHN is tested for its strain sensing behaviors under bending and longitudinal stretching modes. 

## 2. Experimental Section

### 2.1. Materials

Cotton pulp with degree of polymerization (DP), 4500 was purchased from Buckeye Technology, and N/N-Dimethylacetamide (DMAc), zinc nitrate (Zn(NO_3_)_2_), zinc sulfate (ZnSO_4_), triethanolamine (TEA) and ammonium chloride(NH_4_Cl) were purchased from Sigma-Aldrich. 

### 2.2. Fabrication

At first, cellulose film was fabricated by dissolving cotton pulp and regenerating it. Detailed process was previously reported [2,4] and this is a brief summary. The pulp was dissolved by mixing it with DMAc and LiCl mixture at 150 °C with stirring. The cellulose solution was cast on a glass using a doctor blade, followed by putting into a mixture of deionized (DI) water and isopropyl alcohol. And then, a wet cellulose film can be obtained. 

**Figure 1:**
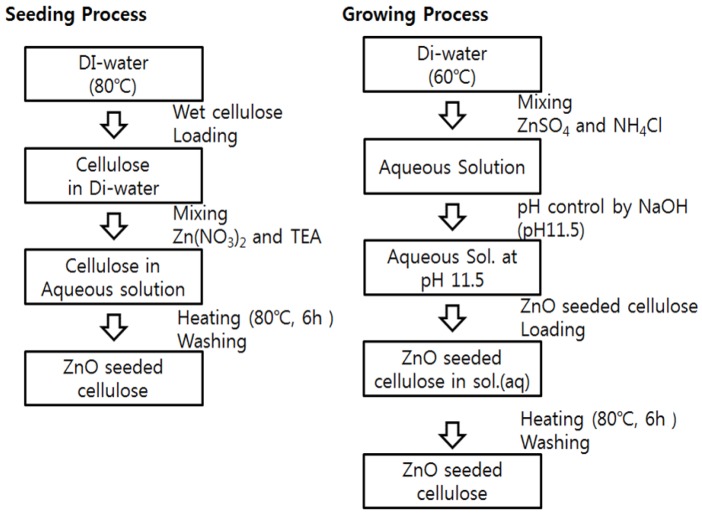
Schematic of CEZOHN (cellulose ZnO hybrid nanocomposite) fabrication process: seeding and growing of ZnO.

ZnO nanorods were grown on the wet cellulose film by using a hydrothermal process. Figure 1 shows the schematic of ZnO growing process divided into seeding and growing. To produce uniform ZnO nanorods, seeding process was added before ZnO nanorods growing. The wet cellulose film was fixed on a glass and it was loaded in 1000 mL DI water at 80 °C. 25 mM Zn(NO_3_)_2_ and TEA were added to the DI water with stirring for 250 rpm. The aqueous solution was stirred and kept at 80 °C for 6 h. After seeding, the cellulose was washed with DI water to remove unfixed ZnO particles and kept in DI water. To grow ZnO nanorods, an aqueous solution of ZnSO_4_ and NH_4_Cl was prepared by adding 15 mM of ZnSO_4_ and 450mM of NH_4_Cl in 500 mL DI water at 60 °C. pH of the solution was controlled to 11.5 with NaOH to lead the deposition of ZnO [16]. The seeded cellulose was loaded in the solution and remained for 6 h at 60 °C. After growing, the ZnO grown cellulose was washed and dried over 6 h in an ambient condition.

### 2.3. Characterization

Morphology of cellulose ZnO was observed by scanning electron microscope (SEM, S-4000, 4300, Hitachi, Tokyo, Japan). X-ray diffraction spectrum has been obtained using XRD (Rigaku, Tokyo, Japan) by using is Cu-Ka1 at 40 kV, 100 mA. FT-IR spectrum (Varian Excalibur FTS 3000, Walnut Creek, CA, USA) was used to analyze the interaction between cellulose and ZnO.

### 2.4. Strain Sensor Test

**Figure 2:**
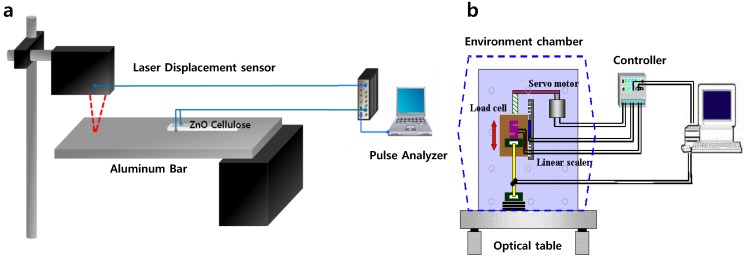
Experimental setup of strain sensor for (**a**) bending mode and (**b**) stretching mode.

Before investigation of strain sensor, piezoelectric charge constant and Young’s modulus was measured by a quasi-static method [2]. The performance of strain senor was tested with bending mode and longitudinal stretching mode. Figure 2 shows the experimental setup of strain sensor for bending and stretching modes. To investigate the strain sensor, aluminum electrode was coated on outsides of cellulose and ZnO layer. The strain sensor was electrically insulated by laminating it with a film. For bending mode test, as shown in Figure 2a, the strain sensor was attached on one side of a fixed aluminum bar (300 × 50 × 2 mm^3^) and tipped at the free end of the bar. Displacement at the free end was measured by a laser displacement sensor (Keyance LK-G85, Tokyo, Japan) and the displacement signal and the generated signal from the attached strain sensor were fed to the pulse signal analyzer. For stretching mode test, the pull test equipment was used as shown in Figure 2b [2]. The strain sensor was fixed between two grippers of the pull test machine and pulling displacement can be precisely controlled. When the strain sensor was pulled, then its displacement and force were monitored from the equipment and induced charge of the strain sensor was measured by using a picoammeter (Keithly 6485, Cleveland, OH, USA). The pulling speed was set to 0.5 μm/s for the quasi-static condition.

## 3. Results and Discussion

### 3.1. ZnO Nanorod Growth on Cellulose

**Figure 3:**
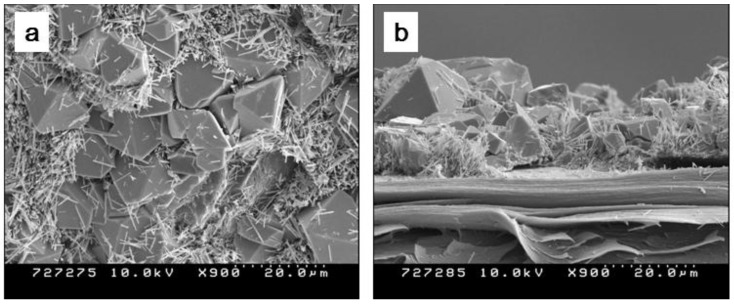
SEM images of CEZOHN without seeding at (**a**) surface and (**b**) cross section.

Before growing ZnO nanorods, the hydrothermal process was conducted without seeding. Figure 3 shows surface and cross sectional SEM images of ZnO nanorods grown on the cellulose film. The cellulose has smooth surface and after the growing ZnO, rod like particles and octahedral ZnO particles fully covered the cellulose surface. In the EDS result, particles are found to be ZnO and/or ZnOH. Octahedral structure of ZnO demonstrates that OH of Zn(OH)_2_ is not perfectly removed by the thermal hydrolysis reaction [16]. To increase the uniformity of ZnO nanorods, additional process is required. 

**Figure 4:**
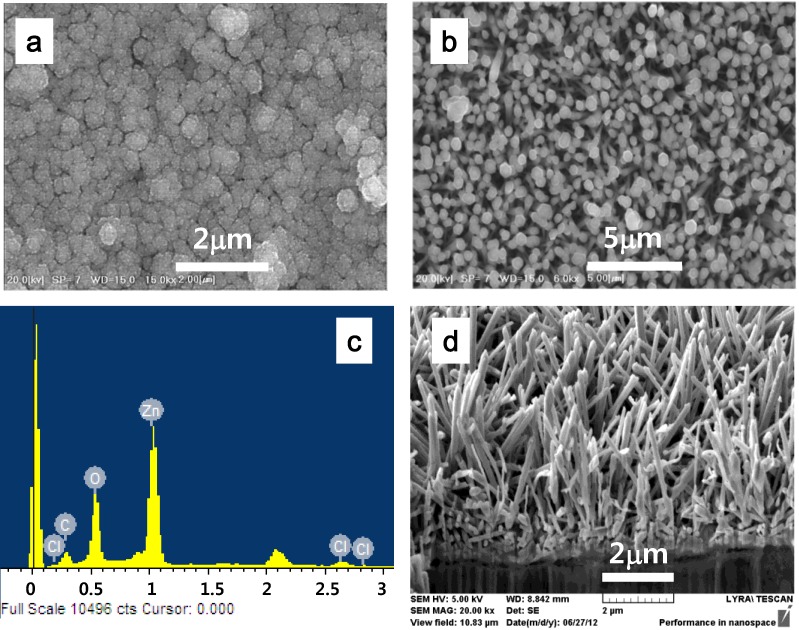
SEM images of CEZOHN with seeding and growing processes: (**a**) seed layer; (**b**) surface; (**c**) EDX and (**d**) cross section.

To uniformly grow ZnO nanorods on the cellulose film, a seeding process was added with the hydrothermal reaction before ZnO nanorod growing. Figure 4a shows the SEM image of ZnO seed layer. The seed layer is composed of 0-demention ZnO spheres. Diameter of ZnO seeds is around 250 nm. ZnO rods are grown on the seed layer by hydrothermal process. Figure 4b shows the surface SEM image of the ZnO nanorods after growing. Diameter of ZnO is similar to ZnO spheres of the seed layer. Figure 4d shows the cross-sectional SEM image of the ZnO nanorods grown on the cellulose film. Figure 4c shows EDS result, which can confirm that ZnO particles are formed.

**Figure 5:**
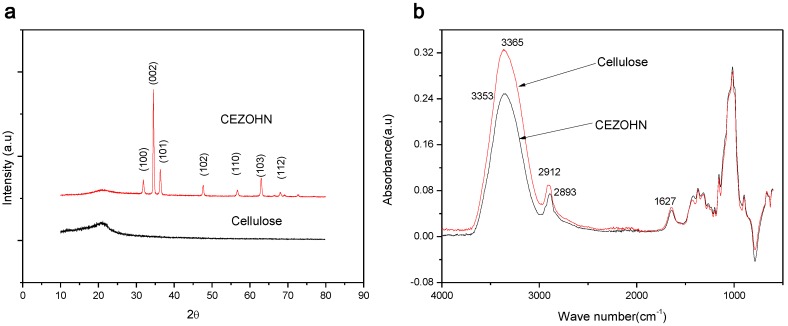
(**a**) XRD result and (**b**) FTIR result of CEZOHN and cellulose.

Figure 5a shows the XRD pattern of CEZOHN. Characteristic peak of cellulose appeared broadly at 2θ = 20.9° and narrow peaks at 31.7°, 34.4°, 36.2° are assigned to (100), (002) and (101), which shows Wurtzite structure of ZnO. Dominant (002) peak indicates that ZnO rods are well aligned perpendicularly, which is shown in Figure 4d. FT-IR spectrum of cellulose and CEZOHN is shown in Figure 5b. At the cellulose spectrum, the broad band of OH functional group is observed at 3365 cm^−1^. The characteristic band of C–C with benzene ring at 1627 cm^−1^ and the C–H vibrations at 2912 cm^−1^ can be attributed the cellulose. Although characteristic band of ZnO is almost covered with cellulose characteristic band in the spectra of CEZOHN, the spectra demonstrate intensity reduction of functional bands of cellulose suggesting that hydroxyl groups are occupied with ZnO. In other words, ZnO is physically interacting to cellulose chains.

### 3.2. Physical Properties

**Figure 6:**
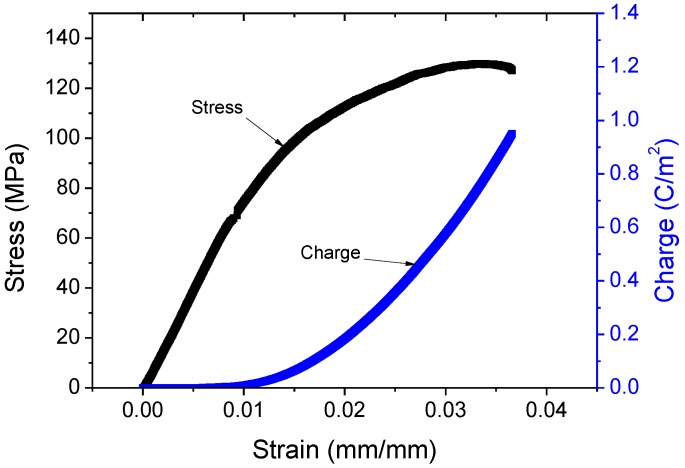
Stress-strain and charge curves of CEZOHN.

To investigate potential of piezoelectric devices, mechanical properties and electromechanical properties of CEZOHN were measured by the quasi-static method. Figure 6 shows the stress-strain curve and induced charge curve. Table 1 shows the comparison of Young’s modulus and piezoelectric charge constant between the aligned cellulose EAPap [17] and CEZOHN. Young’s modulus of CEZOHN is 5.2 GPa, which is almost similar to that of bare cellulose. This result indicates that mechanical rigidity of CEZOHN was not affected by the ZnO growing. Piezoelectric charge constant (*d*_31_) can be calculated from the induced charge (*Q*) and force (*F*) with the following equation [17]:

(1) $$d_{31}=\frac{Q}{A}\frac{tw}{F}$$

where *A* is the electrode area; *t* and *w* are the thickness and the width of CEZOHN. Charge *Q* was found from the measured current. Force *F* was measured by the load cell installed in the pull test equipment. Piezoelectric charge constant of CEZOHN is found to be 145 pC/N. This result is 30 times larger than the bare cellulose and 6 times than the aligned cellulose [18]. We believe that high piezoelectric charge constant of CEZOHN is associated with ZnO nanorods formation on the cellulose film and in-depth study on this phenomenon is on going. 

**Table 1:** Comparison of Young’s modulus and piezoelectric charge constant of cellulose and CEZOHN.

Samples	Young’s modulus (GPa)	Piezoelectric charge constant *d*_31_ (pC/N)
Bare cellulose	5.3	6
Aligned cellulose	7.0	30
CEZOHN	5.0	145

### 3.3. Strain Sensing

**Figure 7:**
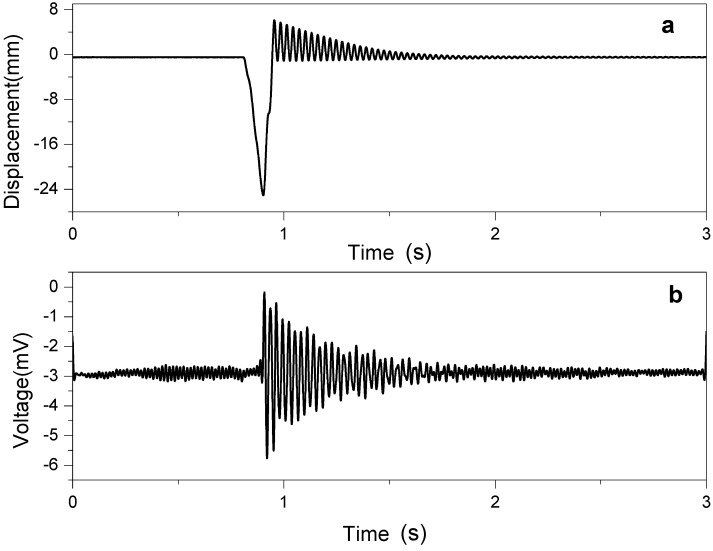
Bending mode strain sensor: (**a**) bending displacement and (**b**) sensor signal.

Strain sensing performance of CEZOHN was tested for bending and longitudinal stretching modes. Figure 7 shows the bending displacement at the tip of the cantilever structure and the strain signal under bending mode. When the cantilever beam was tipped 24 mm, 3 mV of the bending sensor signal was occurred. The voltage signal and displacement signal show an equivalent free vibration. 

**Figure 8:**
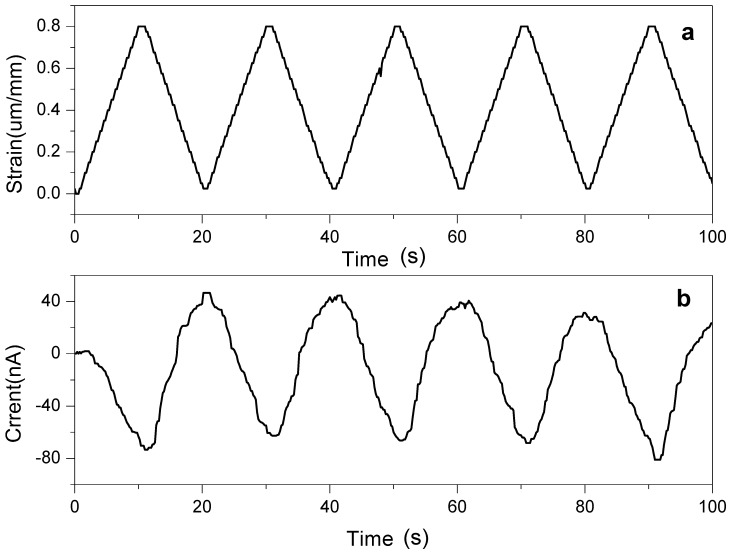
Stretching mode strain sensor: (**a**) stretching strain and (**b**) sensor signal.

The strain sensor for stretching deformation was tested with the pull test equipment and picoammeter. The applied strain is triangular wave form with 0.8 × 10^−3^ peak to peak strain at 0.05 Hz. Figure 8 shows the strain curve and the sensor signal in current. The sensor signal nearly follows the strain curve. As increasing the strain from zero to 0.8 μm/mm, the induced current changed from 40 nA to −80 nA. Sensitivity of the sensor can be found to be 120 nA/0.8 × 10^−3^ m/m = 150 nA/(m/m). From the strain and Young’s modulus, stress σ is E·ε = 5 GPa × 0.8 × 10^−3^ = 40 MPa. Electrical charge can be found by integrating current. So, the maximum charge Q of the first cycle is 0.5 × I × t^2^/A = 0.5 × 120 nA × 10^2^ s^2^/4 × 10^−^^4^ m^2^ = 15 mC/m^2^. Thus, the piezoelectric charge constant *d*_31_ is Q/σ = 375 pC/N.

## 4. Conclusions

Well-aligned ZnO nanorods on cellulose were successfully fabricated by using a low temperature hydrothermal process. Seeding process was introduced for deposition of uniform ZnO nanorods before growing process. Piezoelectric charge constant of CEZOHN was increased 6 times larger than the aligned cellulose case. When the CEZOHN strain sensor was tested under bending and longitudinal stretching modes, it followed the strain curves. In longitudinal stretching mode, calculated piezoelectric charge constant from the sensor signal was similar to the quasi-static piezoelectric test result.
